# Comment on “Using Satellite-Based Spatiotemporal Resolved Air Temperature Exposure to Study the Association between Ambient Air Temperature and Birth Outcomes in Massachusetts”

**DOI:** 10.1289/ehp.1510374

**Published:** 2015-10-01

**Authors:** Cristina Linares, Julio Díaz

**Affiliations:** National School of Public Health, Carlos III Health Institute, Madrid, Spain

In a recently published paper using space-time satellite data to determine air temperature (Ta), Kloog et al. concluded that Ta during pregnancy was associated with lower birth weight and shorter gestational age in the study population. The results obtained pointed to associations between Ta and birth weight during the last trimester, and between Ta and preterm delivery and low birth weight during the entire pregnancy. These results agree with those obtained by Dadvand et al. (2014), indicating Ta has a stressful role influencing low birth weight.

There are many epidemiological studies reporting a nonlinear J- or U-shaped relationship between Ta and health indicators such as mortality (Basu and Samet 2002) and morbidity (Ye et al. 2012), with different response times for heat and cold waves (Gasparrini et al. 2015). When Kloog et al. performed linear and logistic models, Ta was introduced with a linear component in multiple time windows before birth. Models also were adjusted for different environmental and sociodemographic factors. However, the authors did not control for heat and cold episodes during pregnancy. From our point of view, a similar nonlinear behavior could be expected or would at least be worth analyzing in the case of adverse birth variables. As a susceptible population group, pregnant women may be more sensitive to changes in temperature and may be at a greater risk of heat stress, because during pregnancy the increase in fat deposition and associated decrease in the ratio of body surface area to body mass result in less capacity for heat loss to the environment (Wells and Cole 2002).

A study conducted in Rome with time-series analysis methodology (Schifano et al. 2013) showed that heat, as measured by the maximum temperature during warmer periods, was associated in the short term with preterm birth. This association with temperature was not observed in the cold periods. Similar results were obtained in Madrid (Linares et al. in press). This study considered daily maximum temperatures during heat waves (defined in Madrid during 2001–2009 as daily maximum temperatures above 34°C) and during cold waves (defined in Madrid during 2001–2009 as daily maximum temperatures below –2°C). The results for the influence of high temperatures on low birth weight were similar to those found in Rome, with the same short-term association between temperature rise and increased cases of premature birth and, consequently, low birth weight.

This suggests heat waves are an acute stressor on pregnant women, not the chronic stressor described by Kloog et al. The mechanism is unclear by which high temperatures may increase the risk of preterm birth, but there is evidence in the literature that supports a relationship (Carolan-Olah and Frankowska 2014). This short-term effect of Ta on adverse birth outcomes is similar to that already described for traffic noise as a factor in adverse birth outcomes in Madrid (Díaz and Linares in press).

Although Kloog et al. introduced Ta values with linear components, from our point of view introducing Ta as a linear component and not taking into account the effects of heat and cold separately minimizes the authors’ ability to draw conclusions about potential impacts. The effects of high temperatures may be offset by cold temperatures. Therefore, it would be interesting to improve the analysis of Kloog et al. by taking into account the effect of Ta, but separating its effect by warm and cold seasons, especially for days within heat and cold waves.
